# Percutaneous mitral valve repair with the MitraClip NT™ system in a patient presenting with prolonged cardiogenic shock

**DOI:** 10.1002/ccr3.930

**Published:** 2017-09-23

**Authors:** Dominik Buckert, Sinisa Markovic, Markus Kunze, Jochen Wöhrle, Wolfgang Rottbauer, Daniel Walcher

**Affiliations:** ^1^ Department of Internal Medicine II University Hospital Ulm Ulm Germany

**Keywords:** Cardiogenic shock, intra‐aortic balloon pump, Mitral regurgitation, percutaneous mitral valve repair

## Abstract

The MitraClip NT™ system for the treatment of severe mitral valve regurgitation is effective and safe – even for patients suffering from cardiogenic shock. The use of an intra‐aortic balloon pump expands the range of possible applications to this particular group of challenging patients.

## Introduction

Severe mitral valve regurgitation is a common finding in patients with ischemic or nonischemic cardiomyopathy and reduced left ventricular ejection fraction (LVEF). As it often leads to dyspnea and frequently causes or complicates cardiac decompensations in this patient population, indication for mitral valve repair usually is given. The MitraClip NT™ system offers the possibility for an interventional edge‐to‐edge repair in patients who are deemed unsuitable for conventional surgery due to comorbidities and high risk. Nevertheless, current guidelines demand for cardiac stability at the time of the intervention. Consequently, there is only little evidence for the use of this innovative technique in patients suffering from prolonged cardiogenic shock and hemodynamic instability.

## Case History/Examination

**Figure 1:**
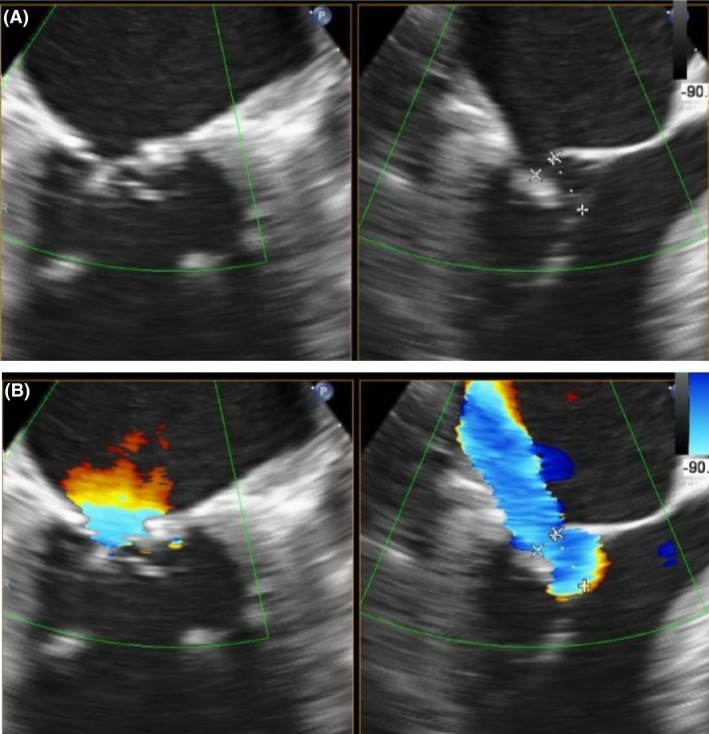
Transesophageal echocardiography showing the native mitral valve before intervention. Two‐chamber view (left) and left ventricular outflow tract view (right) without (A) and with (B) color Doppler encoding. The calcified and restrictive posterior leaflet leading to malcoaptation can be seen.

We report the case of a 78‐year‐old man who was admitted with severe acute cardiac decompensation. The patient exhibited dyspnea at rest (NYHA class IV), as well as central and peripheral edema. Known relevant cardiac conditions were an ischemic cardiomyopathy (state after anterior myocardial infarction) with impaired left ventricular ejection fraction (LVEF ~ 25%), permanent atrial fibrillation, and pulmonary embolism 3 months ago. The diagnostic workup revealed high‐grade mitral valve regurgitation (MR) caused by a severely calcified and restrictive posterior leaflet (segments P2 and P3) with consecutive malcoaptation (Fig. 1). Coronary stenoses or occlusions requiring treatment had been ruled out shortly before the actual admittance. The indication for a cardiac resynchronization therapy was not given in consideration of the narrow QRS complex.

## Treatment

**Figure 2:**
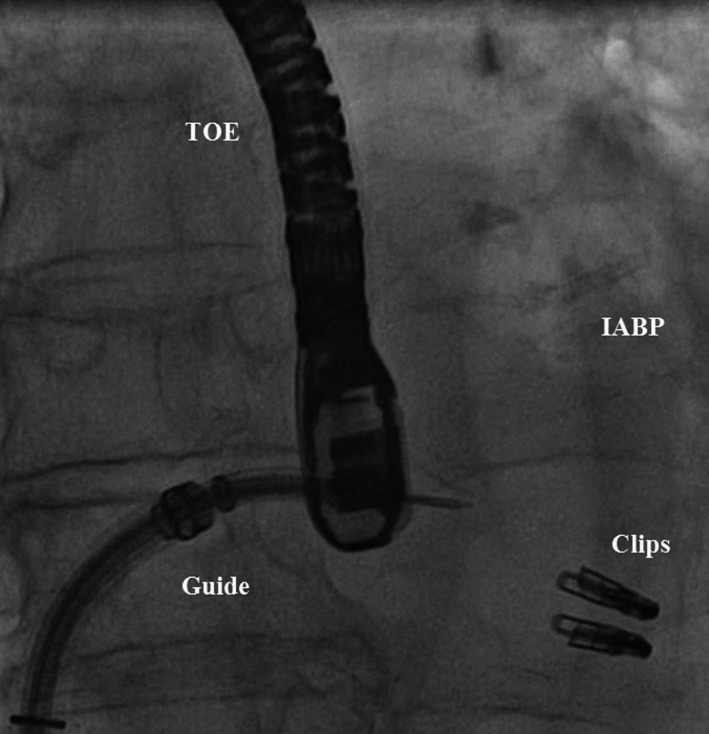
Intraprocedural view: TOE, transesophageal echocardiography; IABP, intra‐aortic balloon pump.

**Figure 3:**
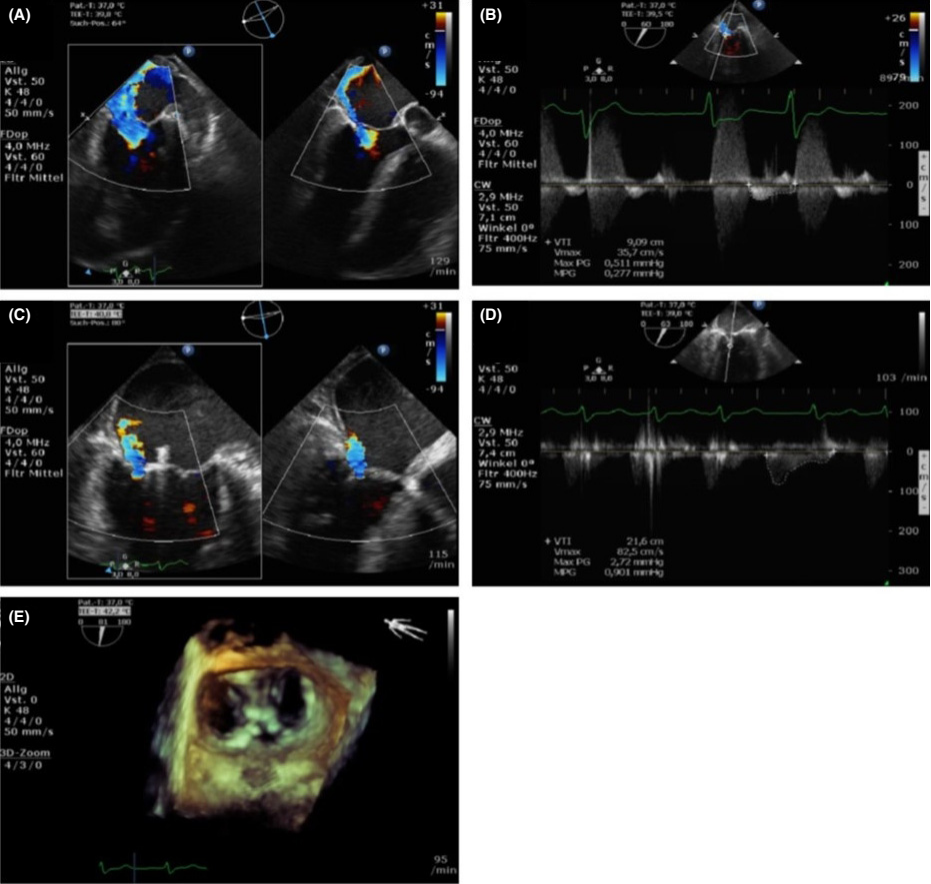
Result of percutaneous mitral valve repair. Comparison of mitral regurgitation before (A) and after (C) clip implantation (transesophageal two‐chamber (left) and left ventricular outflow tract view with color Doppler encoding). Mean transvalvular gradients before (B) and after (D) clip implantation. Surgical view (E) of the mitral valve and the two implanted clips in three‐dimensional reconstruction.

Taking all findings into account, the interdisciplinary heart team decided to treat the mitral valve regurgitation. Because of the unacceptable high risk for a conventional surgical strategy (risk of mortality according to the Society of Thoracic Surgeons risk score: 18.405%; physical status according to the American Society of Anesthesiologists classification: IV), decision was made to perform a percutaneous edge‐to‐edge repair with the new MitraClip NT™ system. Soon after the general anesthesia was applied, rising needs for epinephrine and norepinephrine had to be observed. Despite all efforts, the circulatory situation deteriorated and led to cardiac arrest with pulseless electrical activity. After ten minutes of mechanical resuscitation, spontaneous circulation returned, although the implantation of an intra‐aortic balloon pump (IABP) and the administration of high doses of catecholamines were needed for maintenance. In the absence of alternatives, decision was made to continue the procedure. Because of the complex mitral valve anatomy with a significant malcoaptation gap, we followed a two‐clip strategy (Fig. 2). The first clip was implanted in the medial commissure in order to reduce and stabilize the mobility of the anterior leaflet. The second clip was placed more central at the site of the largest regurgitation (P2/P3). This led to a highly significant MR reduction from grade IV to I (Fig. 3). A relevant increase in the transmitral gradient was not observed (rise of mean gradient from 0.3 mmHg to 1 mmHg). Almost immediately after the deployment of the second clip, catecholamine doses could be reduced by half. The patient was admitted to intensive care unit for further monitoring and therapy.

## Outcome and Follow‐up

Catecholamine infusion could be finished 2 days after the intervention. After another day, the IABP could be removed. Extubation took place at day 4 after the procedure. No neurologic deficits had to be recorded. In the further course, a pleasing improvement in general condition and cardiac situation could be observed. Two weeks after MR intervention, a NYHA II situation could be documented. The patient was re‐evaluated at our outpatient clinic 4 months after discharge. At this time, he presented himself in functional NYHA class II–III. Another cardiac decompensation during this time did not occur. Echocardiography showed a persistent good result with only mild mitral regurgitation. The patient still was on the established heart failure therapy including beta‐blocker, ACE inhibitor, and eplerenone. A relevant adjustment of medication had not been necessary.

## Discussion

The MitraClip NT™ procedure is a well‐established treatment option for patients suffering from high‐grade MR who are at high risk for conventional surgical treatment 1. Nevertheless, current guidelines demand for cardiac stability at the time of the intervention and mention “hemodynamic instability,” “cardiogenic shock,” and “need for IABP” as exclusion criteria 2. As a consequence of these recommendations, there are only few reports on the use of the MitraClip NT™ system as a “rescue option” in patients with cardiogenic shock. In our carefully selected patient, we were able to demonstrate the compelling hemodynamic and clinical consequences following a successful MR intervention. This finding is in concordance with other case reports and thus extends the current evidence base 3, 4. We therefore believe that patients presenting in cardiogenic shock or severe cardiac decompensation should carefully be evaluated concerning the presence of a high‐grade MR and, in case anatomic conditions are suitable, an interventional approach can be offered, even when pharmacological or mechanical circulatory support is needed. As a consequence of our recent experiences, we changed our institutional standard operating procedures. Patients now are evaluated for percutaneous mitral valve repair under established mechanical circulatory support if one or more of the following conditions are given: (1) LVEF<20%, (2) persistent NYHA class IV after adequate medical treatment, and (3) prolonged cardiogenic shock with need for catecholamine administration.

In the present case, the general anesthesia caused a deterioration of the already severely impaired, catecholamine‐dependent circulatory situation, which led to cardiac arrest due to low output and the need for mechanical resuscitation. It remains speculative whether this would have been prevented by an early implantation of the IABP. However, as a consequence of our experiences, we meanwhile follow a more aggressive procedural strategy with an early establishment of mechanical circulatory support in our high‐risk patients.

In principle, implantation of one or more clips can be necessary to achieve a satisfactory MR reduction. There still is controversy on the optimal strategy, especially in the presence of a difficult mitral valve anatomy 5, 6. In cases with a significant coaptation failure, we found following approach being most feasible and leading to best results: By implantation of the first clip next to the main regurgitation site, the functional gap and the mobility of the leaflets generally can be significantly reduced. With the stability gained, the deployment of the second clip in the center of the regurgitation can be facilitated. This strategy opens up the possibility to beneficially use the MitraClip NT™ systems in more complex anatomies and pathologies. In our patient, it led to an excellent MR reduction and to a compelling clinical improvement.

## Authorship

DB: drafted the manuscript, performed literature research, and produced the picture material. SM: helped drafting the manuscript. MK: helped drafting the manuscript. JW: participated in literature research and helped drafting the manuscript. WR: participated in literature research and helped drafting the manuscript. DW: participated in literature research and helped drafting the manuscript.

## Conflict of Interest

None declared.
